# Assessing Quality of Life After Return to Work Among Victims of Work-Related Hand Injuries

**DOI:** 10.1192/j.eurpsy.2025.1877

**Published:** 2025-08-26

**Authors:** A. Haddar, I. Sellami, A. Hrairi, M. A. Ghrab, H. Zouari, A. Feki, M. Trigui, M. Hajjaji, M. L. Masmoudi, K. Jmal Hammami

**Affiliations:** 1 Occupational medicine, University Hospital Hedi Chaker; 2 LR/18/ES-28, University of sfax; 3 Orthopedic, University Hospital Habib Bourguiba; 4 Rheumatology, University Hospital Hedi Chaker, Sfax, Tunisia

## Abstract

**Introduction:**

Work-related hand injuries (WRHI) can have profound impacts on an individual’s physical capabilities, and these injuries often carry long-term consequences that extend beyond physical impairment. Upon returning to work, victims may face challenges in performing occupational tasks and daily activities.

**Objectives:**

This study aims to assess the quality of life in workers who have suffered WRHI after returning to their professional activities.

**Methods:**

We conducted a cross-sectional study among victims of WRHI in the private sector after returning to work, from January 2021 to December 2022. Sociodemographic and professional data were collected along with characteristics of the WRHI. Quality of life was assessed using the Short Form-12 (SF-12) score, which evaluates both physical and mental health components (PCS-12 and MCS-12). The Quick Disabilities of the Arm, Shoulder, and Hand (Quick DASH) score was used to measure the functional disability of the hand. Job satisfaction and pain level were auto-evaluated with a scale of 0 to 10.

**Results:**

We included 126 workers, 88.1% of whom were male, with a mean age of 41.3 ± 10.6 years. Tobacco and alcohol use were reported by 42.9% and 9.5% of participants, respectively, while caffeine consumption was noted in 57.9%. The most represented sectors were metallurgy (22.2%) and the chemical industry (16.7%). The median job satisfaction after the accident was 6 (IQR [5; 8]). In 61.9% of cases, the dominant hand was affected. Both rehabilitation sessions and surgical treatment were required for 69% of participants. The median pain level was 5 (IQR [4; 7]), and 47.6% of participants reported sleep disorders following the accident.

The median Quick DASH score was 34.1 (IQR [13.1; 50.6]), and the median Quick DASH work module score was 43.8 (IQR [25; 68.8]). The mean PCS-12 score was 39.5 ± 7.6, while the mean MCS-12 score was 46.8 ± 11.4. The PCS-12 score was significantly associated with caffeine consumption (p = 0.03), alcohol consumption (p = 0.03), rehabilitation sessions (p = 0.029), and sleep disorders (p < 0.001). It was also significantly correlated with pain level (p = 0.005; r = -0.247), Quick DASH score (p < 0.001; r = -0.4), and the Quick DASH work module (p < 0.001; r = -0.44).

The MCS-12 score was significantly associated with job satisfaction (p = 0.008; r = 0.237), Quick DASH score (p = 0.003; r = -0.265), the Quick DASH work module (p = 0.012; r = -0.23), and sleep disorders (p = 0.012).

**Conclusions:**

Work-related injuries, particularly hand injuries, pose significant challenges to both the professional and personal lives of those affected. Addressing these challenges is crucial to ensuring a successful return to work and social reintegration.

**Disclosure of Interest:**

None Declared

